# Effects of Polysaccharide from *Malus halliana* Koehne Flowers in Cyclophosphamide-Induced Immunosuppression and Oxidative Stress on Mice

**DOI:** 10.1155/2020/1603735

**Published:** 2020-03-12

**Authors:** Yingying Niu, Jing Dong, Huimin Jiang, Jinmei Wang, Zhenhua Liu, Changyang Ma, Wenyi Kang

**Affiliations:** ^1^National R & D Center for Edible Fungus Processing Technology, Henan University, Kaifeng, 475004 Henan, China; ^2^Joint International Research Laboratory of Food & Medicine Resource Function, Kaifeng, 475004 Henan, China; ^3^Kaifeng Key Laboratory of Functional Components in Health Food, Kaifeng, 475004 Henan, China

## Abstract

The immunomodulatory effects of *Malus halliana* flower polysaccharide (MHFP) were investigated in this paper. The model of immunosuppressive mice was established by cyclophosphamide, which was treated with different dosages of MHFP (600, 400, and 200 mg/kg·d^−1^). The results showed that MHFP significantly increased the index of the spleen and thymus and improved the atrophy of immune organs. MHFP enhanced the ability of carbon clearance and phagocytosis of mononuclear phagocytes in mice. Meanwhile, MHFP promoted the proliferation of splenic lymphocytes. MHFP could enhance the content of serum hemolysin and improve the decrease of hemolysin induced by cyclophosphamide. The contents of ACP and LDH in the serum and spleen were determined, indicating that MHFP could enhance the activity of macrophages. MHFP promoted the content of cytokines (IL-2, IL-6, TNF-*α*, and IFN-*γ*) and mRNA expression. At the same time, the pathological changes of the spleen tissue also showed that MHFP could improve the immunosuppression induced by cyclophosphamide. In addition, MHFP increased the content of SOD, T-AOC, and CAT in the serum and spleen tissue, decreased the level of MDA, and improved the oxidative stress caused by cyclophosphamide. In conclusion, MHFP could effectively improve the immunosuppression and oxidative stress induced by cyclophosphamide and enhance the immune capacity of mice.

## 1. Introduction

*Malus halliana* Koehne, belonging to the Rosaceae family, is distributed in Shanxi, Jiangsu, Anhui, and Zhejiang provinces in China [1]. *M*. *halliana* has been used as a traditional Chinese medicine to treat metrorrhagia for a long time [2]. Pharmacological researches showed that *M*. *halliana* had the ability to protect the liver, resisting oxidation, inhibiting *α*-glycosidase *in vitro*, and suppressing the thrombosis *in vivo* [3–5]. Phytochemical research of *M*. *halliana* focused on flavonoids instead of polysaccharides [6, 7].

The body's immune system is composed of immune organs, immune cells, and immune molecules, which can protect against external aggression and protect human health. When the body's immune function is suppressed, it will inevitably be invaded by infection, cancer, and other diseases. In recent years, cancer has become the leading killer of people's health, and immunosuppression is one of the main adverse reactions of drugs to treat cancer [8, 9]. Research and development of immune-enhancing drugs is crucial. It has been reported in the literature that polysaccharides, as a biological macromolecule, have a variety of biological activities. For example, polysaccharides have the functions of antivirus [10], antitumor [11, 12], and antioxidation [13], regulating immunity and maintaining the balance of the immune system [14]. Xin et al. [15] showed that *Flammulina velutipes* polysaccharides could improve the gastrointestinal (GI) function and strengthen the GI peristalsis of patients with constipation. Zhang et al. [16] showed that *F*. *velutipes* polysaccharides could also be used as an immunomodulator. Li et al. [17] and Tang et al. [18] showed that tea polysaccharides and purple sweet potato polysaccharides could regulate the immune system and intestinal microflora. *Dendrobium officinale* polysaccharides also had immunomodulatory activity as well as the potential for a promising dietary therapy for constipation in daily life [19, 20]. Similarly, polysaccharide is one of the active components of *M*. *halliana* flowers, and our previous research proved that the polysaccharide of *M*. *halliana* flowers (MHFP) could effectively improve functional constipation [21]. The results of pre-experiments in our laboratory revealed that MHFP had an immunomodulatory effect. So, in this paper, we evaluated the immunomodulatory effect of MHFP quantificationally and systematically.

## 2. Materials and Methods

### 2.1. Materials and Reagents

Cyclophosphamide was purchased from Jiangsu Hengrui Medicine (Lianyungang, China) Co., Ltd. Indian ink was purchased from Phygene Biotechnology Co., Ltd. Medium RPMI-1640 was purchased from Beijing Solarbio Science & Technology (Beijing, China) Co., Ltd. Concanavalin A (Con A) and lipopolysaccharide (LPS) were purchased from Sigma-Aldrich (USA). Cell Counting Kit-8 (CCK-8) was purchased from Dojindo. Assay kits for total antioxidant capacity (T-AOC), malondialdehyde (MDA), catalase (CAT), superoxidase dismutase (SOD), lactate dehydrogenase (LDH), and acid phosphatase (ACP) were purchased from Nanjing Jiancheng Bioengineering Institute (Nanjing, China). Interleukin-2 (IL-2), interleukin-6 (IL-6), tumor necrosis factor-*α* (TNF-*α*), and interferon-*γ* (IFN-*γ*) were purchased from Nanjing SenBeiJia Biological Technology (Nanjing, China) Co., Ltd.

### 2.2. Polysaccharide of *M. halliana* Flowers

*M*. *halliana* flowers were collected in Henan University (Kaifeng, China) in March, 2017 (the voucher number: 2017032601) and identified by Professor Changqin Li (National R & D Center for Edible Fungus Processing Technology, Henan University). A voucher specimen was deposited in the National R & D Center for Edible Fungus Processing Technology, Henan University. According to our previous paper [22], the dried flowers of *M*. *halliana* (500 g) were extracted with boiling water for three times (1 : 20, 3 h for each time). The combined aqueous extracts were concentrated under reduced pressure at 48°C by rotary evaporator, followed by precipitation in 95% ethanol (final concentration 70%) at 4°C overnight, and then centrifuged at 8000 rpm for 15 min. After centrifugation, the precipitate was dissolved in distilled water and deproteinized 5-10 times by the Sevag method with a mixture of chloroform/1-butanol (4 : 1 *v*/*v*) until protein could not be detected. The protein-free sample was dialyzed in distilled water and precipitated with 95% ethanol (final concentration 70%) at 4°C overnight and centrifuged; the precipitate was redissolved in distilled water and dialyzed for 2 days and lyophilized to obtain crude polysaccharides finally.

### 2.3. Animals and Experimental Design

#### 2.3.1. Animals

Kunming/specifc pathogen-free (KM/SPF) mice (half male and female) weighting 20-22 g were purchased from Henan Animal Experiment Center (with license key SCXK 2017-0004) (Zhengzhou, Henan, China). Mice were housed in cages with unrestricted access to food and water with a constant temperature (25 ± 1°C), relative humidity (45-55%), and a 12 h light/dark cycle. All animals were conducted in accordance with the Guide for the Care and Use of Laboratory Animals of the National Institutes of Health.

#### 2.3.2. Experimental Grouping and Administration

Mice were randomly divided into 5 groups of 10 according to body weight. MHFP high-dose group (MHFP-H), medium-dose group (MHFP-M), and low-dose group (MHFP-L) were, respectively, given 600, 400, and 200 mg/kg MHFP for 21 days by intragastric administration, respectively. The normal group (NG) and model group (MG) were orally administered with normal saline daily for 21 days. And then, MG, MHFP-H, MHFP-M, and MHFP-L received 80 mg/kg cyclophosphamide at day 18, 19, 20, and 21 by intraperitoneal injection, while the NG received normal saline with the same method. After the last administration, mice were fasted for 12 h with free access to water, weighed, and sacrificed by decapitation.

#### 2.3.3. Determination of Thymus Index and Spleen Index

The spleen and thymus were taken, washed with cold saline, and weighed. Then, the spleen index and thymus index of mice were calculated according to the following formulas:

(1) $$\begin{array}{c} \text{Thymus index}=\text{thymus weight }\left(\text{mg}\right)/\text{body weight }\left(10\text{ g}\right),\\ \text{Spleen index}=\text{spleen weight }\left(\text{mg}\right)/\text{body weight }\left(10\text{ g}\right). \end{array}$$

#### 2.3.4. Determination of Clearance Index *K* and Phagocytic Index *A*

Indian ink was injected into the tail vein (10 times diluted) 0.1 mL/10 g, and blood was collected from the orbital sinus and dissolved in 2 mL 0.1% Na_2_CO_3_ solution at 5 min (*t*_1_) and 15 min (*t*_2_), respectively. The absorbance (OD) was measured at 680 nm (OD_1_, OD_2_), and the clearance index *K* was calculated. Mice were sacrificed by decapitation, the liver and spleen were taken, and then phagocytic index *A* was calculated.

(2) $$\begin{array}{c} K=\frac{\log{\text{OD}}_{1}-\log{\text{OD}}_{2}}{t_{2}-t_{1}},\\ A=\sqrt[{3}]{K}\times \frac{\text{Weight}}{\text{Weight of the liver}+\text{Weight of the spleen}}. \end{array}$$

#### 2.3.5. Determination of Hemolysin in Chicken Erythrocytes

On day 7, 5% of saline suspension of chicken erythrocytes was intraperitoneally injected with 0.2 mL. After the last administration, the eyeball blood was collected and centrifuged. The serum was 100 times diluted with normal saline. Then 1 mL of the diluted serum, 0.5 mL of 5% chicken red blood cell suspension, and 0.5 mL of 10% complement were mixed in 37°C for 30 min. And then the reaction was terminated with ice water bath. After centrifugation of the suspension, the absorbance (OD) of the supernatant was measured at 540 nm.

#### 2.3.6. Determination of the Proliferation of Spleen Lymphocytes

Mice were soaked in 75% ethanol for 3 min, and then their spleens were removed in a sterile ultraclean workbench. The spleens were washed with cold PBS and slowly ground through a 200-mesh sieve. The suspension was centrifuged at 1500 r/min for 10 min. The supernatant was discarded, and the precipitate was mixed with the erythrocyte lysate of 3 times volume. After cracking on the ice for 15 min, the precipitate was centrifuged at 1500 r/min for 10 min, abandoned the supernatant, and added 1640 medium to end crack. The crack suspension was centrifuged with 1500 r/min for 10 min. The centrifugal liquid was suspended with 10% fetal bovine serum 1640 medium. The mixture was cultivated at 37°C and 5% CO_2_ cell incubator, and then adherent cells were removed after 24 h. Original generation of spleen cells was stained with trypan blue and counted. The rate of living cells was in 90~93%.

Splenic primary cells were inoculated into 96-well cell culture plates at a rate of 1 × 10^5^ cells per well. LPS (final concentration of 1 *μ*g/mL) or Con A (final concentration of 5 *μ*g/mL) were added to each group. After 68 h of culture, 10 *μ*L CCK-8 was added to each well for 4 h of culture, and then the absorbance (OD) value was measured at 450 nm.

(3) $$\begin{array}{c} \text{Cell proliferation rate }\left(\%\right)=\frac{{\text{OD}}_{\text{Induced}}-{\text{OD}}_{\text{control}}}{{\text{OD}}_{\text{Induced}}}\times 100\%. \end{array}$$

#### 2.3.7. Cytokine Content Determination and mRNA Expression

The blood of mice was taken from the eyeballs, then the blood was stood for 30 min and centrifuged at 4°C with 3500 r/min for 10 min. After the serum was taken, the content of IL-2, IL-6, TNF-*α*, and IFN-*γ* by ELISA kit were determined.

The mice were sacrificed by cervical dislocation and the spleen was taken. The spleen was washed with cold PBS, weighed, and cut into pieces on ice. Then, RNA was extracted and the RNA concentration and purity were determined. The reverse transcription kit was used for reverse transcription of RNA, and the reverse transcribed cDNA was stored at -20°C.

The reverse-transcribed cDNA products were performed by PCR amplification by RT-PCR kit. The GAPDH was used as an internal reference for normalization. The gene expression of IL-2, IL-6, TNF-*α*, and IFN-*γ* was calculated with 2^-*ΔΔ*CT^ method.

#### 2.3.8. Determination of Lactate Dehydrogenase (LDH) and Acid Phosphatase (ACP) and Antioxidative Stress

The serum of mice was taken. The spleen was washed with cold normal saline and weighed. Then, the spleen was cut into pieces on ice, and weight (g): volume (mL) = 1 : 9 of normal saline was added, and was ground thoroughly with tissue homogenizer. The homogenate was centrifuged at 2500 r/min for 10 min, and the supernatant was taken to obtain 10% tissue homogenate. The activities of LDH, ACP, SOD, MDA, CAT, and AOC in the serum and spleen homogenate were determined according to the kit instructions.

#### 2.3.9. Pathological Changes of the Spleen

The spleen was washed with cold saline, fixed with 4% paraformaldehyde for 24 h, washed with PBS for 6 h, dehydrated, embedded, and sliced into 5 *μ*m slices. The slices were stained with HE and examined with an electron microscope.

## 3. Results and Analysis

### 3.1. Effect of MHFP on Thymus Index and Spleen Index

**Figure 1:**
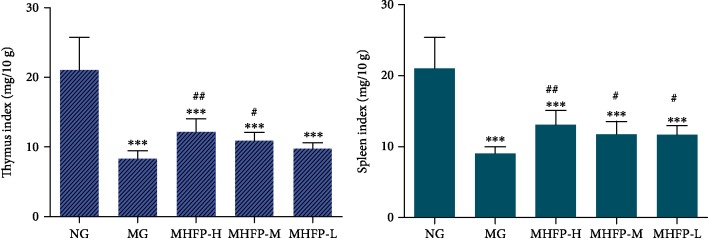
Effect of MHFP on the thymus index and the spleen index in mice (*n* = 10). Compared with the NG: ^∗^*P* < 0.05, ^∗∗^*P* < 0.01, and ^∗∗∗^*P* < 0.001; compared with the MG: ^#^*P* < 0.05, ^##^*P* < 0.01, and ^###^*P* < 0.001.

The spleen and thymus indices of mice were calculated to compare the effects of drug treatment on immunosuppressed mice. In Figure 1, the spleen index and thymus index of the MG were significantly lower than those of the NG (*P* < 0.001). The spleen index of MHFP-H, MHFP-M, and MHFP-L was significantly increased (*P* < 0.05 and *P* < 0.01, respectively) compared with that of the MG, the thymus index of the MHFP-H and MHFP-M were significantly increased (*P* < 0.05 and *P* < 0.01, respectively). The thymus index of MHFP-L was higher than that of the MG, but there was no significant difference.

### 3.2. Effect of MHFP on Clearance Index *K* and Phagocytic Index *A*

**Figure 2:**
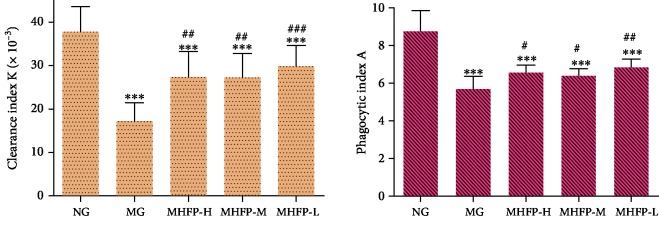
Effect of MHFP on the carbon clearance reaction in mice (*n* = 10). Compared with the NG: ^∗^*P* < 0.05, ^∗∗^*P* < 0.01, and ^∗∗∗^*P* < 0.001; Compared with the MG: ^#^*P* < 0.05, ^##^*P* < 0.01, and ^###^*P* < 0.001.

The carbon particle clearance capacity of mice in each group was calculated according to the above formula. In Figure 2, clearance index *K* and phagocytic index *A* in the MGwere significantly decreased (*P* < 0.001) compared with the NG. The clearance index *K* and phagocytic index *A* were significantly increased in MHFP-H, MHFP-M, and MHFP-L (*P* < 0.05 amd *P* < 0.01) compared with the MG.

### 3.3. Effect of MHFP on Serum Hemolysin Level

**Figure 3:**
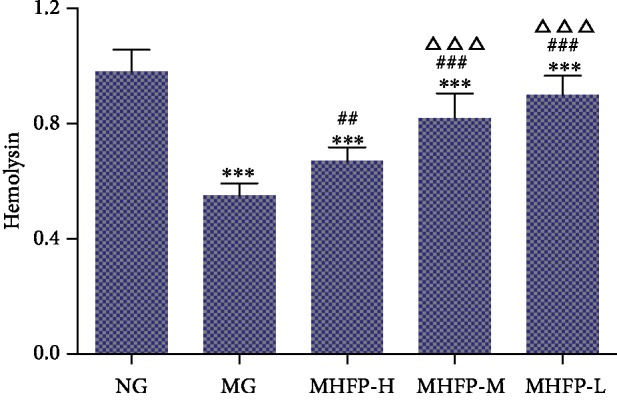
Effect of MHFP on serum hemolysin formation in mice (*n* = 10). Compared with the NG: ^∗^*P* < 0.05, ^∗∗^*P* < 0.01, ^∗∗∗^*P* < 0.001; compared with the MG ^#^*P* < 0.05, ^##^*P* < 0.01, and ^###^*P* < 0.001; compared with the MHFP-H ^△^*P* < 0.05, ^△△^*P* < 0.01, and ^△△△^*P* < 0.001.

The effect of hemolysin in chicken erythrocytes on immune function in mice was observed by intraperitoneal injection. In Figure 3, the serum hemolysin level in the MG was significantly decreased (*P* < 0.001) compared with the NG. The serum hemolysin level in MHFP-H, MHFP-M, and MHFP-L was significantly increased (*P* < 0.01 and *P* < 0.001, respectively) compared with the MG. The serum hemolysin level in MHFP-M and MHFP-L was significantly higher than that of MHFP-H (*P* < 0.001).

### 3.4. Effect of MHFP on the Proliferation of Spleen Lymphocytes

**Figure 4:**
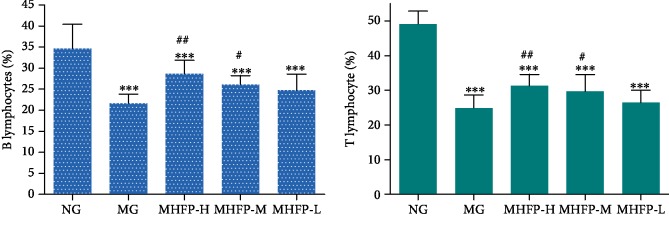
Effect of MHFP on spleen lymphocyte proliferation in mice (*n* = 10). Compared with the NG: ^∗^*P* < 0.05, ^∗∗^*P* < 0.01, ^∗∗∗^*P* < 0.001; compared with the MG: ^#^*P* < 0.05, ^##^*P* < 0.01, and ^###^*P* < 0.001.

Mice spleen lymphocytes were cultured under aseptic conditions. Trypan blue staining was used to calculate the number of living cells. LPS and Con A were used to induce lymphocyte differentiation to observe the effect of MHFP on lymphocyte proliferation in each group. In Figure 4, LPS-induced B lymphocyte proliferation capacity and Con A-induced T lymphocyte proliferation capacity in the MG were significantly lower than those in the NG (*P* < 0.001). Compared with the MG, the proliferation of MHFP-H and MHFP-M was significantly increased, while the proliferation of MHFP-L was increased, but there was no statistically significant difference.

### 3.5. Effect of MHFP on Lactate Dehydrogenase (LDH) and Acid Phosphatase (ACP)

ACP and LDH are markers of macrophage activation. The activities of ACP and LDH were measured to reflect the effect of MHFP on macrophage activity in immunosuppressed mice. In Table 1, the serum ACP and LDH activities in the MG were significantly lower than those in the NG (*P* < 0.01). Compared with the MG, the ACP and LDH activities of MHFP-H and MHFP-M were significantly increased (*P* < 0.05 and *P* < 0.01, respectively). The ACP activity of MHFP-L was significantly increased too. The activity of LDH increased, but there was no difference compared with the MG. In Table 2, the activities of ACP and LDH in the spleen tissues of the MG were significantly lower than those of the NG (*P* < 0.001). Compared with the MG, the activities of ACP and LDH in MHFP-H, MHFP-M, and MHFP-L were significantly increased (*P* < 0.05 and *P* < 0.01, respectively).

### 3.6. Effect of MHFP on the Content of Cytokine

By measuring the content of cytokines in the serum, the effects of MHFP on immune regulation of mice in each group were observed. In Table 3, the serum levels of IL-2, IL-6, TNF-*α*, and IFN-*γ* in the MG were significantly lower than those in the NG (*P* < 0.01 and *P* < 0.001, respectively). Compared with the MG, the serum levels of IL-2, IL-6, TNF-*α*, and IFN-*γ* were significantly higher in the MHFP-H and MHFP-M (*P* < 0.01 and *P* < 0.001, respectively). Compared with the MG, the levels of IL-2, IL-6, and IFN-*γ* were significantly increased in the MHFP-L (*P* < 0.001), and the levels of TNF-*α* were increased without a significant difference.

### 3.7. Effect of MHFP on the mRNA Expression of Cytokines

**Figure 5:**
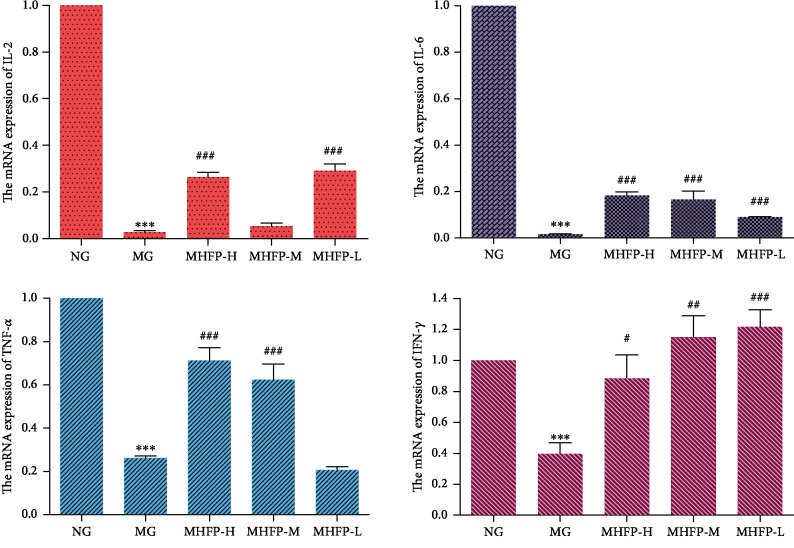
Effect of MHFP on the mRNA expression levels of cytokine in the spleen of mice. Compared with the NG: ^∗^*P* < 0.05, ^∗∗^*P* < 0.01, and ^∗∗∗^*P* < 0.001; compared with the MG: ^#^*P* < 0.05, ^##^*P* < 0.01, and ^###^*P* < 0.001.

Total RNA was extracted from the spleen tissues of mice to determine the expression level of cytokines in the spleen. In Figure 5, the mRNA expression of IL-2, IL-6, TNF-*α*, and IFN-*γ* in the spleen cells of the MG was significantly lower than that of the NG (*P* < 0.01 and *P* < 0.001, respectively). Compared with the MG, the mRNA expression of IL-2, IL-6, TNF-*α*, and IFN-*γ* was significantly increased in the spleen cells of MHFP-H (*P* < 0.05 and *P* < 0.001, respectively). The mRNA expression of IL-6, TNF-*α*, and IFN-*γ* was significantly increased in the MHFP-M and MHFP-L (*P* < 0.05 and *P* < 0.001, respectively), and IL-2 was increased but no significant difference was shown. Compared with the MG, the mRNA expression of IL-2, IL-6, and IFN-*γ* was significantly increased in MHFP-L (*P* < 0.001), and there was no difference in TNF-*α*.

### 3.8. Effect of MHFP on Oxidation Resistance

Cyclophosphamide can cause oxidative stress in the body, and the effect of MHFP on the antioxidative stress ability of immunosuppressed mice was reflected by measuring the levels of SOD, MDA, CAT, and T-AOC. In Table 4, the activities of SOD, CAT, and T-AOC in the serum of the MG were significantly lower than those of the NG (*P* < 0.001), and the content of MDA in the serum of the MG was significantly higher in the NG (*P* < 0.001). Compared with the MG, the activities of SOD, CAT, and T-AOC were significantly increased in the MHFP-H, MHFP-M, and MHFP-L, and the content of MDA was significantly decreased (*P* < 0.001).

In Table 5, the activities of SOD, CAT, and T-AOC in the spleen homogenate of the MG were significantly lower than those of the NG (*P* < 0.01and *P* < 0.001, respectively), and the content of MDA was significantly higher than that of the NG (*P* < 0.001). Compared with the MG, the activities of SOD, CAT, and T-AOC were significantly increased in the MHFP-H, MHFP-M, and MHFP-L. Compared with the MG, the activities of SOD, CAT, and T-AOC were significantly increased in MHFP-H, MHFP-M, and MHFP-L (*P* < 0.05, *P* < 0.01, and *P* < 0.001, respectively), and the content of MDA was significantly decreased (*P* < 0.001).

### 3.9. Effect of MHFP on Pathological Changes of the Spleen Tissue

**Figure 6:**
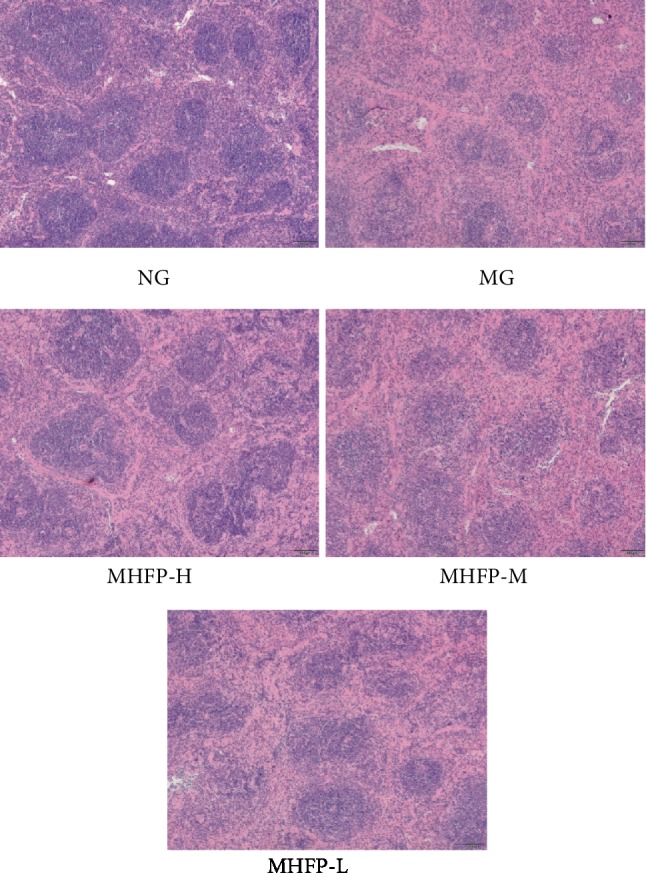
Effect of MHFP on the spleen histological structure in mice.

In Figure 6, the pathological results of HE staining showed that the spleen of mice in the NG had a clear structure, close arrangement of lymphocytes, and the red pulp and white pulp have regular structures and clear edges. In the MG, the white pulp was blurred and the area became smaller, while the lymphocytes were sparse and the number decreased. After administration, the number of lymphocytes in the spleen of mice in MHFP-H was significantly higher than that of the MG, while the lymphocytes were closely arranged and the boundaries of the white pulp were clear. The number of lymphocytes increased and the boundaries of the white pulp were clear in the MHFP-M. The difference in the MHFP-L was small; the number of lymphocytes increased slightly but the boundaries of white pulp was not obvious. The results showed that the MHFP-H could effectively improve the spleen injury caused by cyclophosphamide, while the MHFP-L had no obvious effect.

## 4. Discussion

Cyclophosphamide is commonly used as an antitumor drug with a certain therapeutic effect on a variety of tumors, but this compound cannot show its activity *in vitro*. It would play a role in the immunosuppression and oxidative stress after mainly hydrolyzed by the hepatic cytochrome P450 enzymes [23]. The immune function of the body is performed by a complete immune system consisting of organs, cells, and molecules. The important immune organs include the thymus and spleen [24]. The immune cells include B cells, T cells, and NK cells. The immune molecules include cytokine and immunoglobulin [25].

Immune function is relatively complex, involving all aspects of the body, so the impact of immune function needs to involve many aspects. The spleen is not only a place for the immune cells to settle down but also a place where they can receive antigen stimulation to produce an immune response. The thymus is the site of T lymphocyte differentiation and maturation and is involved in regulating peripheral T cell maturation. The increased weight of the spleen and thymus organs represents proliferation of lymphocytes in the organ, while the decreased immune function may be due to atrophy of the spleen and thymus [26–28]. Thymus and spleen functions can be evaluated by thymus index and spleen index [29]. The previous research indicated that polysaccharide can increase the thymus index and spleen index, and enhance the immune function of mice [30, 31]. The results of this study showed that the thymus index and spleen index of mice increased after MHFP treatment, and the effect is a dose-dependent manner.

Carbon particle clearance method reflects the phagocytic capacity of mononuclear phagocytes. The phagocytic capacity of mononuclear macrophages is one of the important indicators to measure the nonspecific immune function of the body [32, 33]. The results showed that MHFP significantly increased the clearance index and phagocytic index and enhanced the phagocytic ability of mononuclear macrophages in immunosuppressed mice. Antibodies induced by chicken erythrocytes in the body are hemolysin. Hemolysin level can indirectly reflect the production of antibodies, on behalf of the level of antibodies secreted by the body, is an important indicator to detect the humoral immune function of the body [34, 35]. The results showed that MHFP increased the secretion of serum hemolysin and improved the decrease of hemolysin secretion induced by cyclophosphamide. These suggested that MHFP enhanced the humoral immune function of mice.

The activity of acid phosphatase (ACP) and lactate dehydrogenase (LDH) is related to the activation degree of macrophages, which is one of the markers of macrophage activation [36, 37]. Macrophages play an important role in the immune system by phagocytosis of pathogens, presenting antigens and releasing cytokines to participate in various stages of immune response [38, 39]. The contents of ACP and LDH in the serum and spleen tissues were determined in this study. The result showed that the contents of ACP and LDH in the serum and spleen homogenate of MHFP were significantly increased. This suggested that MHFP could enhance the activity of immunosuppressive mouse macrophages.

Lymphocyte proliferation is the most direct indicator reflecting the immune status of the body. The lymphocyte surface has nonspecific mitogen receptors and receptors that recognize antigens such as concanavalin A (Con A) and lipopolysaccharide (LPS). Lymphocytes can proliferate and differentiate when they are stimulated by mitogen *in vitro* or *vivo*, which will increase the synthesis of nucleic acid and protein in lymphocytes, and then be transformed into lymphoblasts with strong metabolism and can be divided. T lymphocyte and B lymphocyte are activated when stimulated by antigen or mitogen. LPS and Con A can induce B lymphocyte and T lymphocyte proliferation, respectively [40]. LPS and Con A were used to induce lymphocyte proliferation, respectively, in our experiment. The results showed that cyclophosphamide inhibited the proliferation of spleen lymphocyte in mice, and MHFP could enhance the proliferation of B lymphocyte and T lymphocyte in immunosuppressed mice, which indicates that MHFP enhanced the immune function of mice by promoting lymphocyte proliferation.

Cytokine is not only an important factor mediating the immune response but also an important factor regulating the immune response. Therefore, to determine the level of the body's immune function, the key is to detect the secretion level of cytokine. Polysaccharides can improve the immune function of mice by activating immune cells and then increasing the level of cytokines in the serum of immunocompromised mice [41, 42]. IL-2 can promote the growth and differentiation of T cells and induce the differentiation of killer cells [43]. IL-6 can regulate the immune responses of T cells and B cells [44]. TNF-*α* is an important cytokine for immune response, and secreted mainly by macrophages [45]. IFN-*γ* promotes T cell differentiation and acts on macrophages to enhance antimicrobial immunity [46]. The results in this paper showed that cyclophosphamide significantly reduced the content and mRNA expression of IL-2, IL-6, TNF-*α*, and IFN-*γ* cytokines in mice, while MHFP could enhance the cytokine secretion and expression of IL-2, IL-6, TNF-*α*, and IFN-*γ*.

Cyclophosphamide can cause oxidative stress and damage the body's normal antioxidant defense system [47, 48]. The destruction of the antioxidant defense system results in the generation of a large number of oxidative intermediates, including free radicals and nonfree radical oxygen-containing molecules, such as superoxide, hydrogen peroxide, and singlet oxygen [49]. SOD, CAT, T-AOC, and MDA are closely related to the body's antioxidant defense system. SOD is a kind of superoxide dismutase, which can catalyze superoxide anion to produce disproportionation reaction and generate hydrogen peroxide [45, 50]. In the cell, hydrogen peroxide is catalyzed by CAT to decompose into H_2_O and O_2_. T-AOC represents the total antioxidant capacity in the body. When the antioxidant system is damaged, excessive lipid peroxides such as MDA will be produced, thus causing cell damage. It has been reported that polysaccharides can enhance the antioxidant activity of immunosuppressed mice [51–53].

Our results showed that MHFP could significantly increase the activities of SOD, CAT, and T-AOC in the serum and spleen tissues, and the levels of MDA in the serum and spleen tissues were decreased in MHFP. The results demonstrated that MHFP could alleviate the oxidative stress induced by cyclophosphamide. The conclusion is consistent with the literature reported.

## Figures and Tables

**Table 1:** Effect of MHFP on activities of LDH and ACP in the serum of mice (*n* = 10).

Groups	ACP	LDH
NG	4.80 ± 0.77	3430.45 ± 484.47
MG	3.20±0.47^∗∗^	2783.82±248.48^∗∗^
MHFP-H	4.28 ± 1.06^#^	3335.57 ± 326.51^##^
MHFP-M	4.33 ± 1.02^#^	3228.26 ± 291.47^#^
MHFP-L	4.32 ± 1.15^#^	2944.16±302.89^∗∗^

Compared with the NG: ^∗^*P* < 0.05, ^∗∗^*P* < 0.01, and ^∗∗∗^*P* < 0.001; compared with the MG: ^#^*P* < 0.05, ^##^*P* < 0.01, and ^###^*P* < 0.001.

**Table 2:** Effect of MHFP on activities of LDH and ACP in the spleen of mice (*n* = 10).

Groups	ACP	LDH
NG	230.65 ± 21.26	3737.22 ± 291.96
MG	165.74±19.88^∗∗∗^	2678.73±442.75^∗∗∗^
MHFP-H	205.30 ± 12.79^∗^^##^	3454.35 ± 187.40^##^
MHFP-M	195.93±13.01^∗∗^^##^	3327.10 ± 115.33^∗^^##^
MHFP-L	192.90±13.52^∗∗^^#^	3257.31 ± 373.42^∗^^#^

Compared with the NG: ^∗^*P* < 0.05, ^∗∗^*P* < 0.01, and ^∗∗∗^*P* < 0.001; compared with the MG: ^#^*P* < 0.05, ^##^*P* < 0.01, and ^###^*P* < 0.001.

**Table 3:** Effect of MHFP on cytokine content in serum of mice (*n* = 10).

Groups	IL-2	IL-6	TNF-*α*	IFN-*γ*
NG	1367.39 ± 73.24	65.31 ± 7.61	230.98 ± 18.49	1580.10 ± 102.07
MG	1134.46±33.39^∗∗∗^	47.17±6.80^∗∗∗^	186.46±18.84^∗∗∗^	1384.96±102.29^∗∗^
MHFP-H	1300.33 ± 80.66^###^	66.14 ± 8.09^###^	231.90 ± 18.04^###^	1549.95 ± 113.67^##^
MHFP-M	1280.22 ± 107.76^∗^^##^	71.18 ± 6.20^###^	219.06 ± 23.64^##^	1642.54 ± 132.66^###^
MHFP-L	1295.33 ± 95.10^###^	89.82±7.23^∗∗∗^^###△△△^	197.43±21.65^∗∗^	1629.34 ± 141.26^###^

Compared with the NG: ^∗^*P* < 0.05, ^∗∗^*P* < 0.01, and ^∗∗∗^*P* < 0.001; compared with the MG: ^#^*P* < 0.05, ^##^*P* < 0.01, and ^###^*P* < 0.001; compared with the MHFP-H: ^△^*P* < 0.05, ^△△^*P* < 0.01, and ^△△△^*P* < 0.001.

**Table 4:** Effect of MHFP on activities of SOD, MDA, T-AOC, and CAT in the serum of mice (*n* = 10).

Groups	SOD	MDA	T-AOC	CAT
NG	792.14 ± 26.44	5.75 ± 1.08	0.5043 ± 0.090	8.04 ± 1.856
MG	638.74±30.49^∗∗∗^	9.82±1.95^∗∗∗^	0.3572±0.047^∗∗∗^	4.16±1.827^∗∗∗^
MHFP-H	701.33±38.48^∗∗∗^^##^	6.08 ± 0.78^###^	0.4402 ± 0.059^#^	6.65 ± 1.780^∗^^###^
MHFP-M	687.62±26.53^∗∗∗^^#^	6.27 ± 1.29^###^	0.4680 ± 0.060^##^	6.78 ± 1.308^###^
MHFP-L	691.18±72.92^∗∗∗^^##^	6.80 ± 1.10^###^	0.4289 ± 0.061^∗^^#^	6.17±1.539^∗∗^^##^

Compared with the NG: ^∗^*P* < 0.05, ^∗∗^*P* < 0.01, and ^∗∗∗^*P* < 0.001; compared with the MG: ^#^*P* < 0.05, ^##^*P* < 0.01, and ^###^*P* < 0.001.

**Table 5:** Effect of MHFP on the activities of SOD, MDA, T-AOC, and CAT in the spleen of mice (*n* = 10).

Groups	SOD	MDA	T-AOC	CAT
NG	383.5761 ± 16.66	5.627 ± 0.86338	0.1803 ± 0.033	6.3510 ± 1.52
MG	292.6363±39.02^∗∗^	7.732±0.52281^∗∗∗^	0.0966±0.012^∗∗∗^	2.8643±0.57^∗∗∗^
MHFP-H	372.2228 ± 40.73^##^	5.7809 ± 0.71231^###^	0.1308±0.009^∗∗∗^^##^	5.2996 ± 1.35^∗^^###^
MHFP-M	368.2792 ± 40.16^##^	6.0197 ± 0.35613^###^	0.1216±0.007^∗∗∗^^#^	4.4419±0.94^∗∗∗^^###^
MHFP-L	346.6108 ± 28.61^#^	6.0456 ± 0.60381^##^	0.1121±0.005^∗∗∗^	4.1848±1.10^∗∗∗^^##^

Compared with the NG: ^∗^*P* < 0.05, ^∗∗^*P* < 0.01, and ^∗∗∗^*P* < 0.001; compared with the MG: ^#^*P* < 0.05, ^##^*P* < 0.01, and ^##^*P* < 0.001.

## Data Availability

The [DATA TYPE] data used to support the findings of this study are included within the article.
